# Relationship between muscle strength and rehospitalization in ventricular assist device patients

**DOI:** 10.1038/s41598-021-04002-3

**Published:** 2022-01-07

**Authors:** Kiyonori Kobayashi, Masato Mutsuga, Akihiko Usui

**Affiliations:** 1 Department of Rehabilitation, Nagoya University Hospital, 65 Tsurumai-cho, Showa-ku, Nagoya, Aichi 466-8560 Japan; 2 Department of Cardiac Surgery, Nagoya University Graduate School of Medicine, 65 Tsurumai-cho, Showa-ku, Nagoya, Aichi 466-8560 Japan

**Keywords:** Cardiac device therapy, Health care

## Abstract

We examined the relationship between leg extensor muscle strength (LEMS) at discharge and rehospitalization within 1 year in patients with a newly implanted ventricular assist device (VAD). This study included 28 patients who had received a VAD at our institution between October 2013 and February 2019, all of whom had been discharged for 1 year. The patients were divided into two groups according to their LEMS at discharge (higher strength [group H] and lower strength [group L]), based on the median value of the 55.2 kg-force (kgf)/body weight (BW) equation. Exercise performance parameters (e.g., grip strength, 6-min walk distance, and peak VO_2_) and laboratory data concerning nutritional status were also collected. Nine patients (64.3%) in group L were rehospitalized within 1 year after discharge. The rehospitalization rate was significantly higher in group L than group H (*p* = 0.020). Compared with discharge, patients exhibited higher grip strength (56.3 vs. 48.6 kg/BW, respectively; *p* = 0.011), 6-min walk distances (588 vs. 470 m, respectively; *p* = 0.002), and peak VO_2_ (15.4 vs. 11.9 mL/min/kg, respectively; *p* < 0.001) at 1 year after discharge. However, the LEMS (57.4 vs. 58.0 kgf/BW, respectively; *p* = 0.798) did not increase after discharge in VAD patients who avoided rehospitalization. LEMS at discharge was associated with rehospitalization after VAD surgery; a high LEMS improves the likelihood of avoiding rehospitalization.

## Introduction

According to the Interagency Registry for Mechanically Assisted Circulatory Support (INTERMACS), the rehospitalization rate during the 3 years after ventricular assist device (VAD) implantation surgery is 64–75%; causes of rehospitalization include infection, bleeding, equipment trouble, and right heart failure^1–3^. Among the adverse events leading to rehospitalization, infection and bleeding are associated with body mass index (BMI) at the time of VAD surgery^4^. In VAD patients, low BMI and low albumin level at discharge are risk factors for rehospitalization^5^. Therefore, proper management of nutrition and physical function is necessary. Previous investigations of skeletal muscle in VAD patients have demonstrated differences in prognosis according to the muscle mass, as measured on preoperative computed tomography scans; however, no reports have examined the rehospitalization rate according to clinically useful parameters (e.g., muscle strength). For postoperative management of VAD patients, muscle strength constitutes a valuable index. Exercise training during inpatient rehabilitation for recovery of physical function focuses on leg extensor muscle strength (LEMS). However, because few reports have examined the recovery course of LEMS itself, it is unclear whether LEMS affects the prognosis of VAD patients^6^. This study examined the relationship between LEMS at discharge and the rate of rehospitalization within 1 year in patients with a newly implanted VAD. To our knowledge, this is the first report concerning the importance of LEMS in VAD patients during the early to middle postoperative period.

## Methods

The participants were 28 VAD (HeartMate II LVAD; Thoratec Corporation, Pleasanton, California, USA) patients who had been discharged home from our hospital between October 2013 and February 2019. Data were collected on patient age, sex, etiology, ultrasound physiological test results, and blood biochemical test results. Measurements were conducted at and after discharge. Measurements after discharge were based on the evaluation score at the time of “educational hospitalization” after initial discharge. The educational hospitalization involved checking the patient’s compliance with self-management and providing educational guidance. Additionally, catheterization was performed to adjust the continuous flow rate of the pump. To distinguish between planned educational hospitalization and hospitalization due to an adverse event, the latter was considered as the need for > 7 days of medical treatment.

The evaluation indices for physical function were grip strength and LEMS^7^. Grip strength was measured in a sitting position with the elbow at 90° flexion and the forearm in a neutral position; measurements were conducted in the second grip position of the grip meter (Jamar, Clifton, NJ, USA); values were adjusted for body weight (BW)^8^. LEMS was measured using a hand-held dynamometer with the knee joint at 90° flexion in a sitting posture; values were again adjusted for BW^9^. Exercise tolerance was evaluated based on the 6-min walk distance and maximum oxygen intake (peak VO_2_)^10^. To examine the relationship between rehospitalization and LEMS, patients were divided into two groups based on the median LEMS at discharge (higher strength [group H] and lower strength [group L]). To compare patient factors between the groups, the unpaired *t*-test, Mann–Whitney *U* test, and chi-squared test were used. The rate of rehospitalization within 1 year after discharge was analyzed using Kaplan–Meier survival curves. Additionally, changes in physical function and exercise tolerance after discharge were investigated in patients who were not rehospitalized due to adverse events within 1 year after discharge. Changes in indices between discharge and educational hospitalization were assessed using the paired t-test and Wilcoxon signed-rank test. Relationships between continuously distributed variables were examined by linear regression analysis; Pearson’s correlation analysis was also performed. Stepwise multiple regression analysis was used to assess independent associations among variables. IBM SPSS Statistics software (version 26.0: IBM Corp., Armonk, NY, USA) was used for the statistical analysis. *P* < 0.05 was considered to indicate statistical significance. This study was approved by the Nagoya University School of Medicine Ethics Committee (approval number: 2019–0272). All procedures were conducted in accordance with relevant guidelines and regulations. The need for informed consent was waived by the Nagoya University School of Medicine Ethics Committee. All information was collected retrospectively from medical records.

## Results

### Difference in leg extensor muscle strength between the time of discharge and rehospitalization

**Figure 1:**
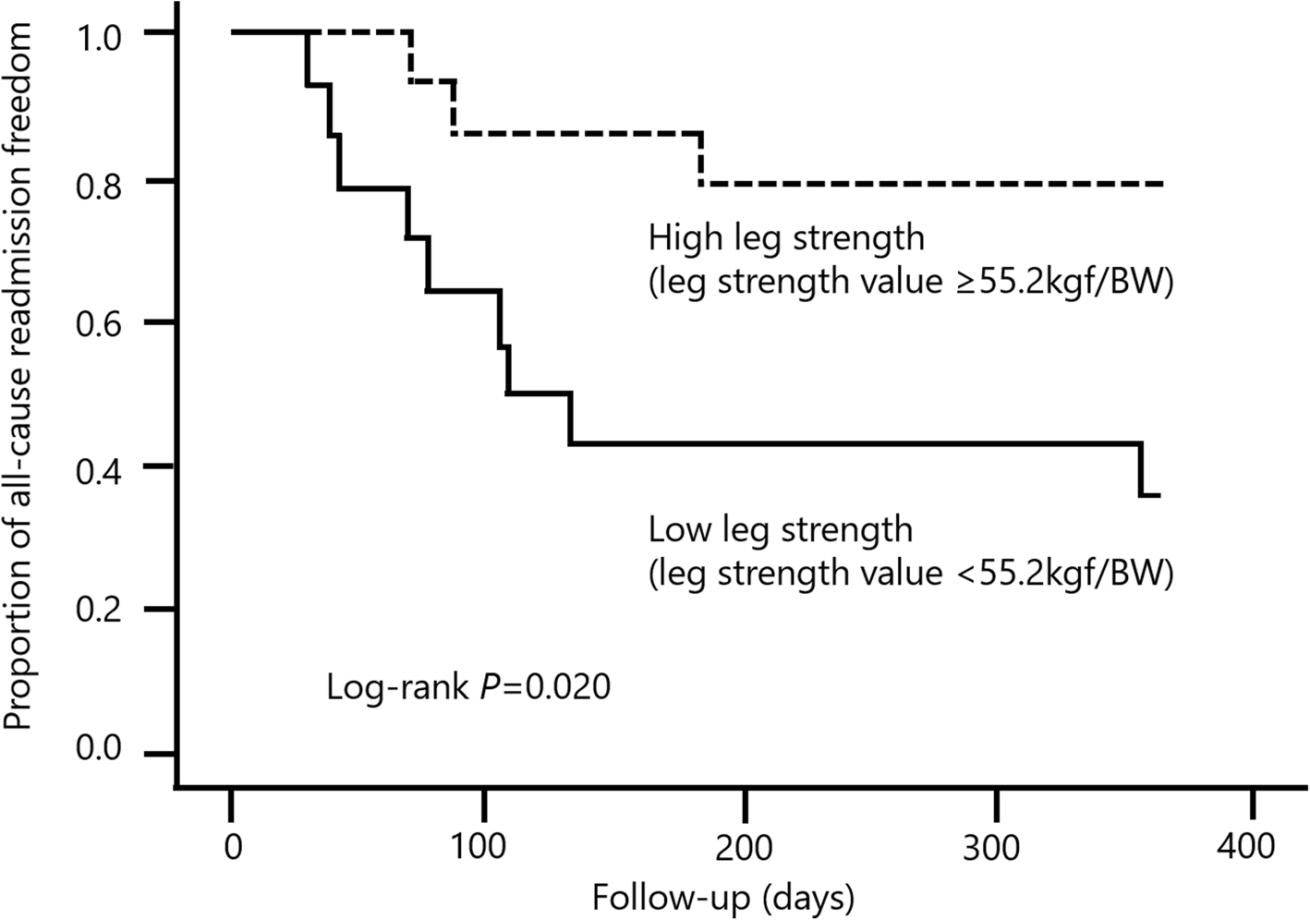
Relationship between leg strength at discharge and the rate of rehospitalization within 1 year of discharge.

The median LEMS value among the 28 left VAD patients at discharge was 55.2 kg-force (kgf)/BW. The participants were divided into higher-strength (≥ 55.2 kgf/BW, group H) and lower-strength groups (< 55.2 kgf/BW, group L), as stated above, according to the LEMS at discharge. Of the 28 patients, 12 were rehospitalized within 1 year after discharge (42.9%; infection, *n* = 6; bleeding, *n* = 3; dislocation, *n* = 2; heart failure, *n* = 1). There were significantly more rehospitalizations due to adverse events in group L than group H (*n* = 9, 64.3% vs. *n* = 3, 21.4%; log-rank, *p* = 0.020) (Fig. 1).

**Figure 2:**
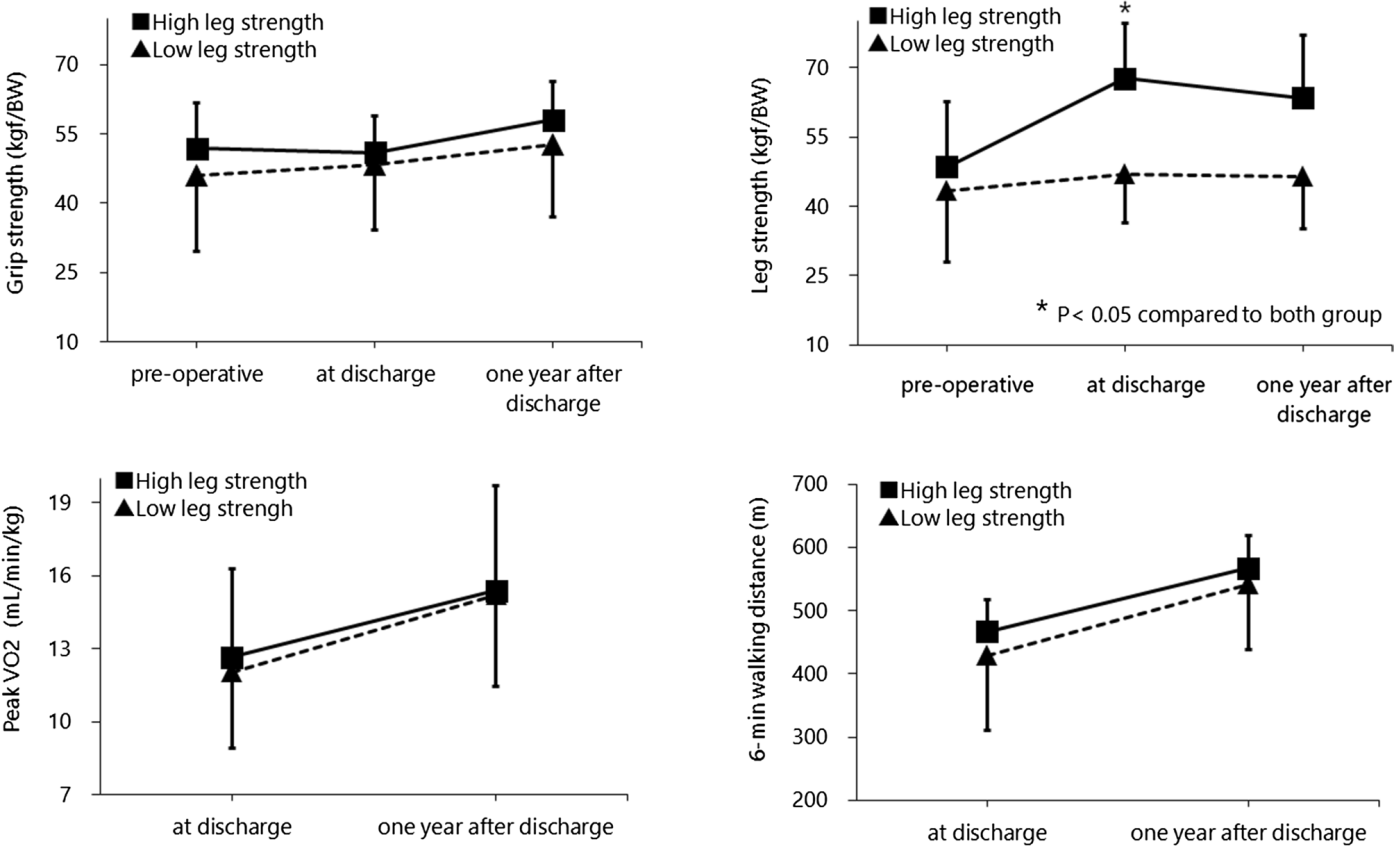
Comparison of physical function between the high and low leg strength groups.

There were no deaths within 1 year of discharge. There were no differences in age (*p* = 0.250), BMI (*p* = 0.944), left ventricular ejection fraction (*p* = 0.564), serum creatinine (*p* = 0.835), C-reactive protein (*p* = 0.475), brain natriuretic peptide (*p* = 0.805), grip strength (*p* = 0.919), 6-min walking distance (*p* = 0.296), or peak VO_2_ (*p* = 0.956) between the groups (Tables 1, 2, 3, Fig. 2).

**Table 1:** Preoperative characteristics.

Overall (*n* = 28)	High leg strength (*n* = 14)	Low leg strength (*n* = 14)	*p-*value				
Age (years)	44.4 ± 14.1	46.9 ± 12.0	0.250				
Men/women	12/2	11/3	0.500				
Etiology of heart failure			0.738				
Dilated cardiomyopathy	9	10					
Ischemic cardiomyopathy	3	2					
Others	2	2					
INTERMACS profile			0.649				
1	3	4					
2	2	3					
3	9	6					
4	0	1					
Strategy used for device implantation			1.000				
Bridge to transplant	11	11					
Bridge to bridge	3	3					

	Median	IQR	Range	Median	IQR	Range	*p*-value
Intensive care unit period before surgery (days)	15	37.8	0–314	17	30.3	0–246	0.925
Time from hospitalization to surgery (days)	82	86.3	31–345	83	52.5	27–292	0.854
Preoperative BMI (kg/m^2^)	20.4	4.8	16.2–26.5	20.0	3.3	14.9–23.8	0.458
Preoperative physical function							
Grip strength (kgf/BW)	52.4	7.3	30.4–65.8	41.2	17.1	21.0–82.6	0.124
Leg strength (kgf/BW)	51.3	24.3	28.0–69.2	39.0	19.8	22.5–72.0	0.411

*INTERMACS* Interagency Registry for Mechanically Assisted Circulatory Support, *IQR* interquartile range, *BMI* body mass index, *BW* body weight.

**Table 2:** Comparison of characteristics between the high and low leg strength groups at discharge.

Overall (*n* = 28)	High leg strength (*n* = 14)			Low leg strength (*n* = 14)			*p-*value
	Median	IQR	Range	Median	IQR	Range	
Postoperative length of stay (days)	82	85.5	52–324	99	60.5	61–282	0.800
Echocardiography at discharge							
LVEF (%)	27.4	24.0	7.6–49.0	23.4	9.0	4.6–46.5	0.564
LVDd (mm)	56.3	16.7	37.9–86.3	55.2	16.6	32.2–77.0	0.880
LVDs (mm)	48.5	21.7	32.0–81.6	50.3	16.3	28.3–74.0	0.967
Laboratory data at discharge							
Serum albumin (g/dL)	3.8	1.0	2.4–4.8	3.9	0.8	2.1–4.4	1.000
Serum creatinine (mg/dL)	0.85	0.21	0.48–1.38	0.77	0.29	0.51–1.58	0.835
Serum sodium (mEq/L)	139	3	137–142	140	3	137–143	0.209
Aspartate aminotransferase (IU/I)	20	5	13–33	20	7	13–34	0.445
Total bilirubin (mg/dL)	0.6	0.3	0.4–1.5	0.8	0.4	0.4–1.6	0.463
C-reactive protein (mg/dL)	0.20	0.44	0.07–11.26	0.43	0.43	0.04–4.41	0.475
Plasma BNP (pg/mL)	181.3	229.2	40.7–836.7	141.0	267.7	24.7–853.8	0.805
BMI (kg/m^2^) at discharge	20.8	2.9	16.4–26.2	19.5	2.8	16.1–24.1	0.944
Rate of change in BMI (%)	101.6	6.7	79.8–114.9	97.2	11.0	83.7–109.0	0.415
Physical function at discharge							
Grip strength (kgf/BW)	51.9	10.9	38.3–62.5	44.0	10.8	22.9–75.9	0.919
Rate of change in grip strength (%)	97.6	25.6	76.7–140.3	106.7	12.5	91.5–141.1	0.168
Leg strength (kgf/BW)	63.9	16.6	55.4–90.1	51.4	7.1	18.3–54.9	0.024
Rate of change in leg strength (%)	132.6	93.2	82.2–321.9	130.4	69.4	72.0–204.8	0.218
Peak VO_2_ (mL/kg/min)	11.7	3.1	8.9–23.3	12.0	5.1	7.5–16.6	0.956
6-min walking distance (m)	465	70	310–600	440	206	230–600	0.296

*IQR* interquartile range, *BW* body weight, *BMI* body mass index, *LVEF* left ventricular ejection fraction, *LVDd* left ventricular diastolic diameter, *LVDs* left ventricular systolic diameter, *BNP* brain natriuretic peptide.

**Table 3:** Comparison of data between the high and low leg strength groups at 1 year after discharge.

Overall (*n* = 28)	High leg strength (*n* = 14)			Low leg strength (*n* = 14)			*p-*value
Readmission rate, *n* (%)	3 (21.4)			9 (64.3)			0.022

	Median	IQR	Range	Median	IQR	Range	*p-*value
Echocardiography after discharge							
LVEF (%)	20.6	19.9	8.0–54.8	27.3	18.3	3.6–47.0	0.663
LVDd (mm)	59.8	20.1	35.0–89.0	63.6	21.4	46.3–92.5	0.735
LVDs (mm)	51.7	23.3	25.3–85.7	58.3	27.0	38.7–91.0	0.909
Laboratory data after discharge							
Serum albumin (g/dL)	4.4	0.5	3.1–5.0	4.2	0.5	2.8–4.7	0.943
Serum creatinine (mg/dL)	0.93	0.32	0.52–1.81	0.87	0.20	0.54–1.22	0.889
Serum sodium (mEq/L)	139	2	135–141	140	1	136–144	0.227
Aspartate aminotransferase (IU/I)	21	10	13–42	26	7	13–41	0.136
Total bilirubin (mg/dL)	0.8	0.1	05–2.5	0.8	0.5	0.4–1.4	0.139
C-reactive protein (mg/dL)	0.10	0.21	0.03–0.52	0.27	0.23	0.03–4.03	0.627
Plasma BNP (pg/mL)	120.5	235.1	9.5–496.6	113.1	159.7	9.4–733.5	0.618
BMI (kg/m^2^) after discharge	23.9	5.6	18.8–30.1	21.3	3.6	16.0–29.9	0.002
Physical function after discharge							
Grip strength (kgf/BW)	57.9	11.1	44.2–74.2	49.3	18.3	27.0–81.6	0.568
Leg strength (kgf/BW)	59.4	9.2	43.9–94.5	47.6	13.4	23.4–62.1	0.180
Peak VO_2_ (mL/kg/min)	14.7	4.3	10.8–25.0	15.3	4.6	9.6–21.6	0.428
6-min walking distance (m)	552	100	489–651	548	83	345–715	0.182

*IQR* interquartile range, *BW* body weight, *BMI* body mass index, *LVEF* left ventricular ejection fraction, *LVDd* left ventricular diastolic diameter, *LVDs* left ventricular systolic diameter, *BNP* brain natriuretic peptide.

**Figure 3:**
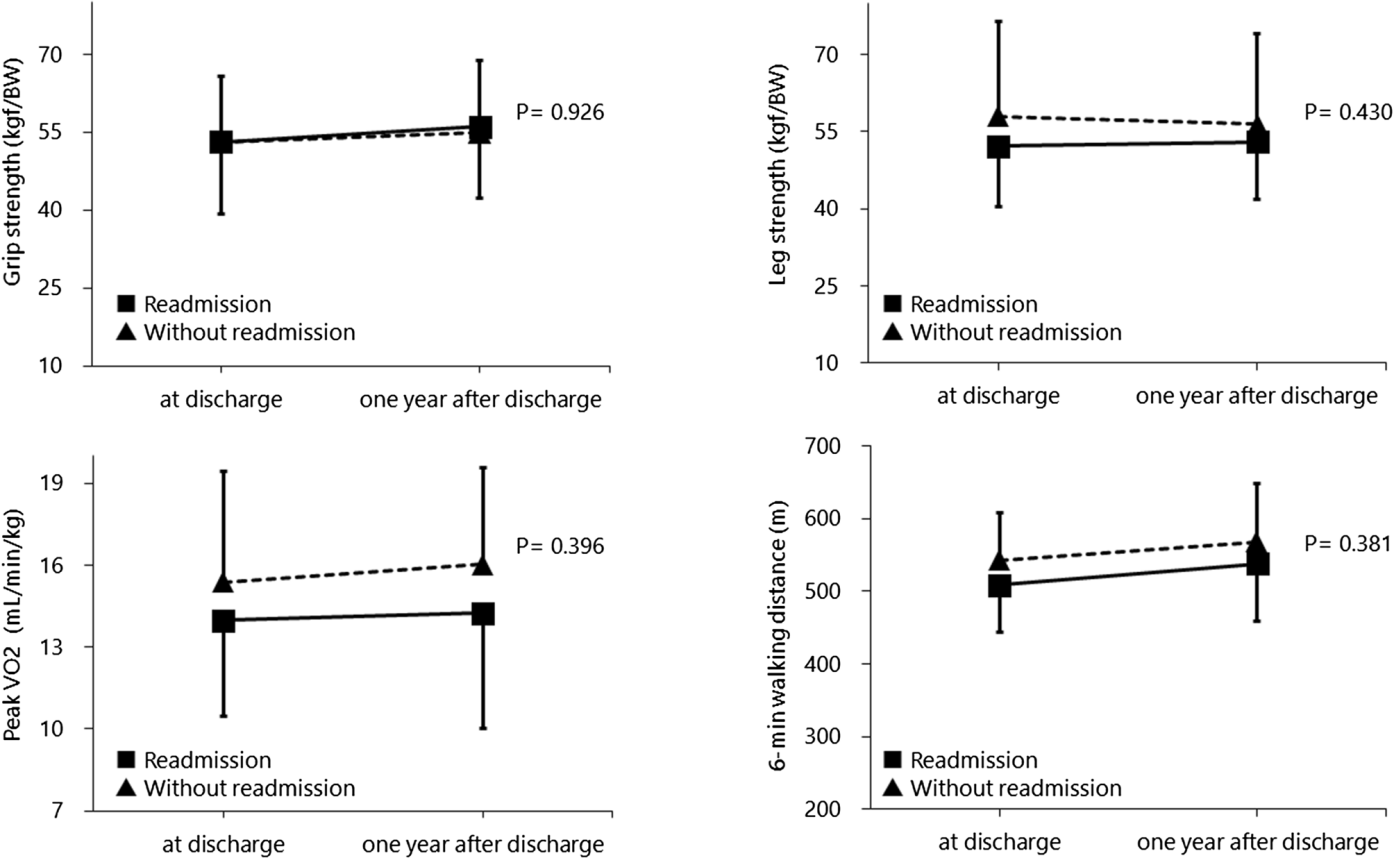
Comparison of physical function between patients with and without readmission.

In addition, there were no differences in physical function between the 12 rehospitalized patients and the 16 patients who were not rehospitalized (Fig. 3).

Multiple regression analysis was performed using a forward stepwise approach; variables that were significantly associated with LEMS at discharge in the univariate analysis were included. Total bilirubin (*P* = 0.006), brain natriuretic peptide (*P* = 0.020), and aortic regurgitation severity (*P* = 0.038) were independently correlated with LEMS at discharge (Table 4).

**Table 4:** Stepwise liner regression analysis of variables associated with leg muscle strength in VAD patients (*n* = 28).

Total bilirubin		BNP		AR	
SC	*p*-value	SC	*p*-value	SC	*p*-value
–0.408	0.039	–	–	–	–
–0.442	0.017	0.403	0.028	–	–
–0.492	0.006	0.397	0.020	0.353	0.038

*SC* standard coefficient, *BNP* brain natriuretic peptide, *AR* aortic regurgitation severity.

### Changes in physical function after discharge

Because changes in physical function are affected by rehospitalization, we investigated changes in physical function between the time of discharge and 1 year after discharge in the 16 patients who avoided rehospitalization. The physical function data at 1 year after discharge were derived from measurements obtained at the time of educational hospitalization. Comparisons of data obtained at discharge and 1 year thereafter revealed significant changes in BW (*p* = 0.004), BMI (*p* < 0.001), serum albumin (*p* = 0.003), serum creatinine (*p* = 0.017), total bilirubin (*p* = 0.028), and C-reactive protein (*p* = 0.008) (Table 5).

**Table 5:** Changes in physical function in left VAD patients who avoided rehospitalization.

Patients without readmission (*n* = 16)	At discharge			One year after discharge			*p*-value
	Median	IQR	Range	Median	IQR	Range	
BW (kg)	59.4	6.4	49.2–83.4	66.5	22.3	56.0–90.2	< 0.001
BMI (kg/m^2^)	20.2	3.5	16.4–26.2	23.9	7.4	18.1–30.1	0.004
Echocardiography							
LVEF (%)	23.4	15.4	4.6–44.8	17.3	14.2	3.6–53.9	0.863
LVDd (mm)	69.4	19.7	41.0–86.3	68.9	20.3	46.8–92.5	0.089
LVDs (mm)	62.8	22.4	32.0–81.6	62.5	21.6	35.6–91.0	0.137
AR (0/I/II/III/IV)	(1 / 10 / 3 / 0/ 0)			(2 / 7 / 4 / 1/ 0)			0.763
Mitral regurgitation severity (0/I/II/III/IV)	(4 / 5 / 5 / 0/ 0)			(6 / 2 / 5 / 1/ 0)			1.000
Laboratory data							
Serum albumin (g/dL)	3.8	0.8	2.4–4.7	4.3	0.4	3.5–4.9	0.003
Serum creatinine (mg/dL)	0.78	0.20	0.53–1.12	0.84	0.16	0.54–1.20	0.017
Serum sodium (mEq/L)	139	4	137–142	139	2	136–141	0.816
Aspartate aminotransferase (IU/I)	20	5	13–34	23	9	16–41	0.211
Total bilirubin (mg/dL)	0.6	0.6	0.4–1.6	0.8	0.4	0.4–2.5	0.028
C-reactive protein (mg/dL)	0.40	0.91	0.07–11.26	0.17	0.25	0.03–2.39	0.008
Plasma BNP (pg/mL)	194.6	319.8	24.7–476.7	99.3	255.7	9.4–450.1	0.158
Physical function							
Grip strength (kgf/BW)	48.6	11.9	22.9–75.9	56.3	17.2	27.0–80.8	0.011
Leg strength (kgf/BW)	58.0	12.4	18.3–83.0	57.4	15.5	23.4–94.5	0.793
Peak VO_2_ (mL/kg/min)	11.9	3.7	8.9–23.3	15.4	5.8	10.8–25.0	< 0.001
6-min walking distance (m)	470	160	350–600	588	100	400–715	0.002

*VAD* ventricular assist device, *IQR* interquartile range, *BW* body weight, *BMI* body mass index, *LVEF* left ventricular ejection fraction, *LVDd* left ventricular diastolic diameter, *LVDs* left ventricular systolic diameter, *AR* aortic regurgitation severity, *BNP* brain natriuretic peptide.

**Figure 4:**
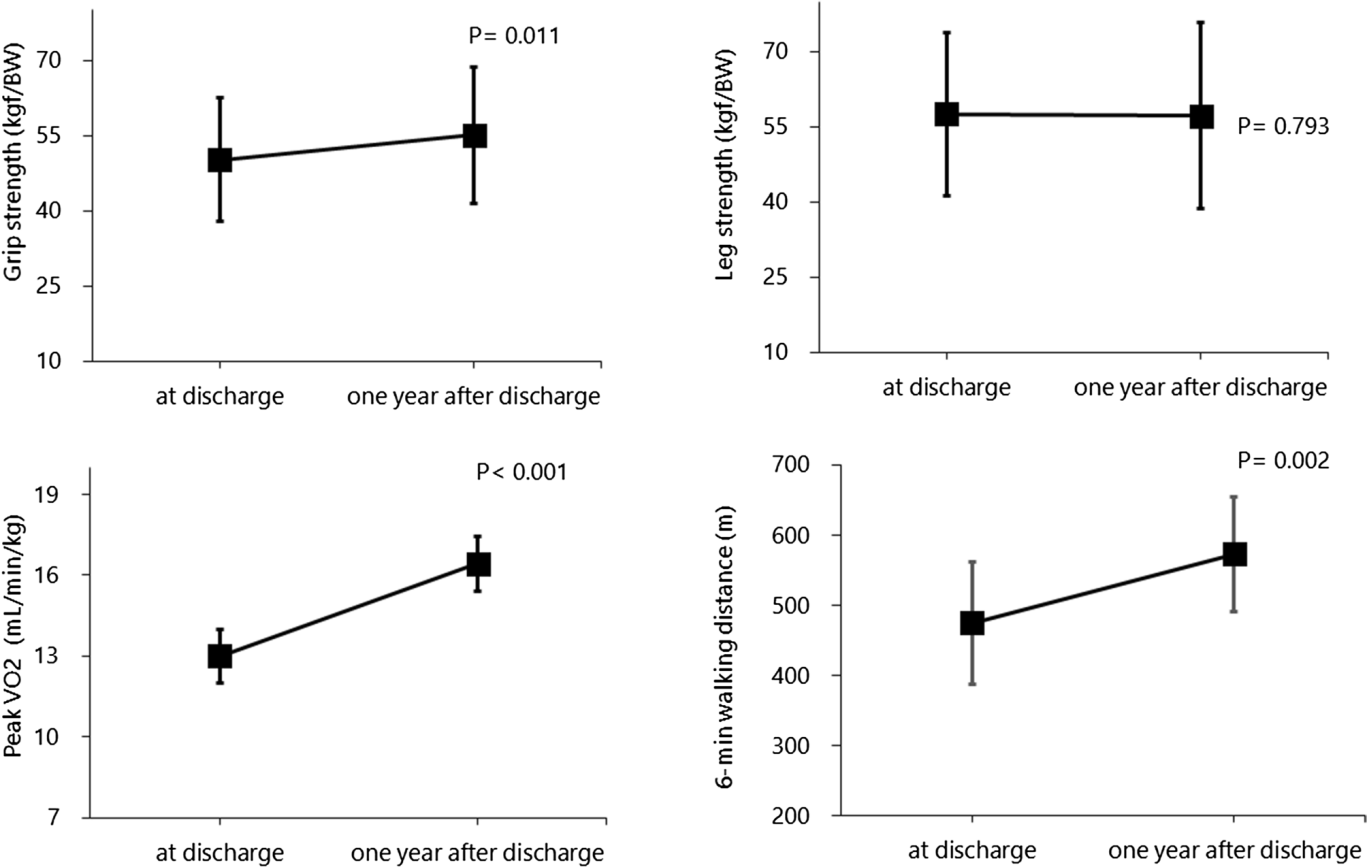
Comparison of physical function between discharge and 1 year after discharge in patients who avoided rehospitalization.

With respect to physical function, significant changes were observed in grip strength (*p* = 0.011), peak VO_2_ (*p* < 0.001), and the 6-min walking distance (*p* = 0.002), although the difference in LEMS was not statistically significant (57.5 ± 16.3 kgf/BW at discharge vs. 57.2 ± 18.5 kgf/BW at 1 year after discharge; *p* = 0.798) (Fig. 4).

## Discussion

This study investigated the relationships of LEMS at discharge with rehospitalization and physical function in VAD patients during the early to middle postoperative period after discharge. The rate of rehospitalization within 1 year was higher in group L than group H. The 1-year rehospitalization rate was 64.3% in group L, nearly identical to the rate (64%) in a previous study^11^. Previous studies have not reported an association between rehospitalization and LEMS; thus, the association between LEMS at discharge and the rehospitalization rate within 1 year reported in the present study was a novel finding. Multiple regression analysis showed that brain natriuretic peptide, total bilirubin, and aortic regurgitation severity were independently associated with LEMS at discharge. LEMS at discharge is a surrogate marker of the degree of recovery of general condition; it may be affected by preoperative heart failure. Rehospitalization due to adverse events has been associated with measures of nutritional status (e.g., BMI) before VAD surgery, and with both serum albumin and BMI at discharge^5,12^. In addition, a study that focused on perioperative grip strength showed increases in long-term mortality in patients with low grip strength after VAD use^13^. These reports suggest that prolongation of perioperative cardiac cachexia is a risk factor for rehospitalization and mortality. Previous studies have reported a connection between frailty and adverse events^5,14^. Although our participants were less frail than those in previous studies, the degree of recovery from general wasting may have been affected by the extent of heart failure management. Changes in LEMS and BMI from before surgery to the time of discharge did not differ between our groups. Several studies have shown that a combination of exercise and nutritional therapy improved cardiac cachexia, because protein synthesis-mediated recovery from muscle wasting was necessary^15–17^. Rehospitalization due to adverse events is a risk factor for perioperative muscle wasting, due to the lack of opportunity for vigorous muscle training.

Grip strength is an indicator of muscle strength. However, in cardiac surgery patients, upper limb muscle training interventions (e.g., grip strength) have not been implemented in daily clinical practice because of the need to avoid unwanted bone adhesion. The response of lower limb skeletal muscle to exercise training is considered an indicator of recovery in clinical practice. Lower limb muscle strength is reportedly a prognostic indicator in frail patients with non-VAD heart failure^18^. However, non-left VAD patients may experience worsening heart failure due to heavy exercise; thus, vigorous resistance training is not appropriate for this group. Discharged VAD patients are the most likely group to benefit from exercise therapy because they can begin vigorous resistance training^19^. We consider lower limb muscle strength an indicator of the improvement potential of VAD patients. In this study, leg muscle strength was associated with readmission, and thus could be a target for rehabilitation.

The LEMS did not change in the middle postoperative period in this study. Body surface area reportedly affects patient outcomes after VAD implantation^20,21^. The rate of change in BMI from before surgery to discharge tended to be lower in group L. Patient BMI at discharge in this study was similar to that in the J-MACS study^20^, but was much lower than the BMI reported for VAD patients in Europe and the United States^7^. BMI affects recovery from frailty^22^. The lack of increase in LEMS among the patients in this study presumably resulted from long-term incapacitation, combined with low physical activity and malnutrition.

In the early to middle postoperative period, grip strength and exercise tolerance were higher compared with discharge. However, the LEMS did not change. BMI on admission for patients undergoing educational hospitalization was higher than that of patients who were not rehospitalized. LEMS did not change in the middle postoperative period, but BW might have increased. This is similar to the results of a previous study, in which BMI increased 2 years after surgery^23^. Because the present study used a retrospective observational design, causality was difficult to establish. Wearing a VAD has an impact on daily life^24^. Resistance training prevents adverse events in patients with cardiovascular disease^25^. Muscle weakness involves insufficient protein synthesis; thus, our patients with low muscle strength may have had a higher rate of readmission due to both the lack of vigorous resistance training and protein synthesis.

Reports from other countries have shown that grip strength improves over time, which accords with our results^13^. Our results for peak VO_2_ were also similar to those of previous studies^10,26,27^. Heart transplantation typically involves a long period of wearing mechanical assistance devices before the actual transplant, such that a strategy for maintaining patient quality of life during this period is needed^28^. Patients with severe heart failure commonly show skeletal muscle dysfunction^29^; it is difficult to preserve skeletal muscle function in daily life during long-term mechanical circulation support. LEMS reportedly increases in heart failure patients during the recovery phase due to the effects of cardiac rehabilitation^30^. Future studies should observe physical functioning in patients in the context of long-term mechanical support; home management strategies should be established^31^. To maintain quality of life, patients must maintain their physical function. Therefore, clinicians should be attentive to physical function when patients are hospitalized. Our facility does not provide supervised outpatient rehabilitation. Previous studies have reported the usefulness of cardiac rehabilitation for left VAD patients^32^. However, the infrequent implementation of outpatient cardiac rehabilitation is a problem in Japan^33^. In particular, continuous medical care for VAD patients is limited to dedicated VAD management facilities; inpatient training is important because of the challenges presented by outpatient cardiac rehabilitation. Furthermore, LEMS should be a target for rehabilitation interventions, although our study did not clearly demonstrate that patients with low leg strength show marked improvement after intervention. Additional investigations are needed to further explore the potential for strength recovery of VAD patients.

## Limitations

Two patients in group H and three in group L lacked lower limb strength data before surgery. Measurement of lower limb muscle strength cannot be performed in patients unable to sit unassisted. Therefore, these data were lacking for patients who underwent intra-aortic balloon pump surgery during intensive care unit management.

Additionally, this retrospective observational analysis of the second-generation HeartMate II (Thoratec Corporation, Pleasanton, California, USA) axial-flow device included a small number of cases; therefore, the hazard ratio for rehospitalization could not be calculated using multivariate regression. In the future studies, we plan to include more cases involving next-generation devices, such as the continuous-flow HeartMate III (Thoratec Corporation, Pleasanton, California, USA) device, to investigate the relationship between physical function and prognosis. Our ultimate goal is to maximize the potential of these devices.

As a final limitation, we only considered the early to middle postoperative period. Leg extensor muscles are prone to atrophy in severe heart failure patients, but the course of leg muscle strength recovery in the middle to late postoperative period is unclear. Thus, recovery during the middle to late postoperative period should be investigated.

## Conclusions

Differences in LEMS at discharge may influence the likelihood of rehospitalization after VAD surgery. Because the LEMS of VAD patients is unlikely to increase after discharge, it is important to focus on cardiac rehabilitation during hospitalization with the goal of attaining adequate LEMS.
